# The Effectiveness of Mindfulness-Based Interventions for Depressive Symptoms and Their Relationship to Interoceptive Awareness: A Systematic Review

**DOI:** 10.31083/AP39860

**Published:** 2025-12-23

**Authors:** Samantha Knep, Alice Shires

**Affiliations:** ^1^Psychology Department, University of Technology, Sydney, NSW 2007, Australia

**Keywords:** mindfulness, depression, cognitive behavioural therapy, adult

## Abstract

**Background:**

This systematic review aimed to investigate the effectiveness of mindfulness-based interventions (MBIs) in treating depression, enhancing interoceptive awareness (IA), and whether IA mediates this relationship.

**Methods:**

In August 2024, a comprehensive literature search was conducted in web-based medical and psychological databases, including PsycINFO, MEDLINE, and Scopus, following the Preferred Reporting Items for Systematic Reviews and Meta-Analyses guidelines. Studies were included if they were quantitative, peer-reviewed, in English, used MBIs derived from Mindfulness-based Stress Reduction (MBSR), Mindfulness-based Cognitive Therapy (MBCT), or Mindfulness-integrated Cognitive Behavioural Therapy (MiCBT), included a control/comparison group, pre- and post-intervention measures, assessed depressive symptoms and IA in adults over 18, and had at least 20 participants. Exclusion criteria included non-English publications, dissertations, case studies, qualitative research, therapies not derived from the specified MBIs, and studies with under 20 participants or individuals under 18. Methodological quality and risk of bias were assessed.

**Results:**

Six studies involving 646 participants met the inclusion criteria. All MBIs (MBCT, MBSR, MiCBT, Mindfulness-based Cancer Recovery, and Mindful Awareness in Body-Oriented Therapy) significantly reduced depressive symptomology and improved IA across varying effect sizes, with IA identified as a partial mediator.

**Conclusions:**

MBIs appear to alleviate depressive symptoms and improve IA, with one study finding IA as a mediator. Limitations included limited literature, search term specificity, heterogeneity and mixed evidence quality. Future research should explore IA’s mediating role, develop a standardised IA measure, and integrate IA into broader treatment modalities to enhance outcomes.

**The PROSPERO Registration:**

CRD42023457300, https://www.crd.york.ac.uk/PROSPERO/view/CRD42023457300.

## Main Points

1. Mindfulness-based interventions (MBIs) demonstrate efficacy in reducing depressive symptoms and enhancing interoceptive awareness (IA) across varied clinical populations.

2. Preliminary findings support the therapeutic relevance of IA, particularly self-regulation and body listening, in the context of MBIs.

3. While IA is considered a potential mechanism of change, only one included study directly examined its mediating role, indicating a gap in the current evidence base.

4. The substantial heterogeneity in intervention protocols, outcome measures, and participant populations limits the generalisability of the findings.

## 1. Introduction 

Depression is a common and often recurrent mental health condition that significantly impacts individuals’ wellbeing and functioning. Mindfulness-based interventions (MBIs) are gaining momentum in research and therapy for their potential to enhance mental wellbeing and health outcomes [1, 2]. MBIs incorporate mindfulness techniques, which have shown promise in alleviating depressive symptoms and preventing relapse, though the underlying mechanism(s) remain under investigation [3, 4]. Contemporary research suggests that interoceptive awareness (IA) may be central to MBIs’ therapeutic effects, given its role in emotion regulation and equanimity, the ability to remain unperturbed by experiences within the framework of the body and mind [5, 6]. Therefore, this review aims to synthesise the literature on MBIs’ effectiveness in reducing depressive symptoms and enhancing IA and examine IA’s potential as a mediator in this relationship.

### 1.1 Mindfulness

Mindfulness has gained significant popularity in the scientific literature over the past two decades [1]. Originating around 500 BC in Buddhist India, mindfulness is a meditation practice that emphasises present-moment awareness to foster discernment and insight [7]. Modern definitions often define mindfulness as a state of consciousness involving attentional regulation and a non-judgemental attitude towards current experiences, including thoughts, feelings, and bodily sensations [8, 9, 10]. Widely adopted in Western culture, mindfulness has been shown to enhance cognitive performance, improve attention span, and reduce stress, depression and anxiety [1, 2]. These benefits have led to its integration into therapeutic settings, resulting in the development of MBIs.

### 1.2 Mindfulness-Based Interventions

MBIs are typically concise, consisting of eight to ten sessions, and can be delivered individually or in groups [10]. Research indicates that MBIs yield favourable outcomes across various psychopathologies [2, 11], physical health conditions, and non-clinical challenges [10, 12]. By using mindfulness principles, MBIs enhance mental health and wellbeing through present-moment awareness [1] and contemporary mindfulness meditation practices, helping participants recognise bodily sensations and develop equanimity and emotion regulation strategies [6]. They focus on transforming the relationship with thoughts to prevent their proliferation, promoting mindful acceptance of challenging experiences and cultivating metacognitive awareness with acceptance and compassion [13, 14].

Strauss *et al*. [10] identify Mindfulness-Based Stress Reduction (MBSR) [15] and Mindfulness-Based Cognitive Therapy (MBCT) [16] as two of the more robustly appraised and extensively developed MBIs. A more recent addition to this field is Mindfulness-integrated Cognitive Behaviour Therapy (MiCBT) [5].

#### 1.2.1 Mindfulness-Based Stress Reduction

MBSR is recognised as a leading MBI and has been the foundation for many subsequent approaches [4]. Designed for individuals with chronic medical conditions, MBSR is a structured 8-week group program rooted in traditional Buddhist mindfulness meditation practices [17]. It includes weekly 2-hour sessions, 45 minutes of daily mindfulness practice, and a full-day silent retreat in week 6 [4, 18]. MBSR aims to alleviate stress and improve quality of life through breath awareness, body scanning meditation, and yoga [9]. It emphasises stress reduction by integrating mindfulness practices into daily activities, fostering emotional balance and regulation [4, 19].

#### 1.2.2 Mindfulness-Based Cognitive Therapy

MBCT, adapted from MBSR, was developed to prevent relapse in individuals with major depressive disorder [4, 20]. This structured, manualised program includes an 8-week course featuring weekly 2-hour group meditation sessions and daily guided home practices [21, 22]. MBCT combines MBSR’s mindfulness practices with elements of cognitive therapy to enhance awareness of automatic thoughts and habitual reactions to negative stimuli [23]. Research posits that MBCT helps reduce rumination and promote more adaptive cognitive strategies by decentring from negative thoughts [24]. Moreover, MBCT has shown effectiveness for individuals with recurrent depression [25].

#### 1.2.3 Mindfulness-Integrated Cognitive Behavioural Therapy

MiCBT is a structured, manualised second-generation transdiagnostic MBI that integrates mindfulness meditation with Cognitive Behavioural Therapy (CBT) [5, 26]. Delivered over an 8-to-10-week program, it utilises Socratic questioning, behavioural experiments, exposure techniques, and daily mindfulness meditation [13]. MiCBT enhances awareness and equanimity by focusing on bodily sensations and emphasising the interplay between thoughts and body sensations. It fosters non-reactive IA through mindfulness and body scanning practices [26]. A key feature is its focus on the cognitive and semantic aspects of internal experiences, which can reinforce behaviour and help individuals manage their reactions to internal signals and thoughts [27, 28].

#### 1.2.4 Mindfulness-Based Interventions and Depression

Research increasingly supports MBIs as effective in reducing depressive symptoms and preventing relapse [3, 4, 8, 29]. Studies consistently show that MBIs enhance self-awareness and emotional regulation, significantly alleviating depressive symptoms compared to controls [2, 10, 30]. For example, MBSR demonstrates moderate effectiveness across diverse populations, including significant symptom reduction in older adults. Research highlights MBCT’s effectiveness in reducing depressive symptoms, especially in major depressive disorder, often yielding larger effect sizes than MBSR [24, 31]. Meta-analyses confirm MBCT’s efficacy across varying depressive episode histories [32]. Additionally, MiCBT shows promise for reducing depressive symptoms in conditions such as diabetes, multiple sclerosis, and alcohol misuse [33, 34, 35]. While evidence supports the effectiveness of MBIs in reducing depressive symptoms, limitations remain. Many studies rely on self-report measures, potentially introducing bias, and sample characteristics often vary widely, limiting generalisability.

### 1.3 Interoceptive Awareness 

The specific mechanism(s) producing positive outcomes in MBIs remains uncertain. IA has been identified as an essential element in the emotion-regulatory system and may represent a key factor in the effectiveness of mindfulness [36]. IA involves the perception of internal bodily sensations, such as satiety, heartbeat, respiration, and autonomic nervous system activities linked to emotions [1, 37]. Conscious awareness of these sensations is known as IA, which Fissler *et al*. [6] describe as the ability to perceive and mentally represent the body’s physiological state. Chen *et al*. [38] further suggest that IA involves the nervous system’s ability to detect, interpret, and manage internal body information [39].

Measuring IA is challenging due to varied definitions and broad applications. Khalsa *et al*. [40] describe IA as an umbrella term encompassing various facets of interoception, complicating the isolation and assessment of specific components. This complexity is compounded by diverse self-report measures such as the Multidimensional Assessment of Interoceptive Awareness (MAIA) [41], the Body Perception Questionnaire (BPQ) [42] and the Five Facets of Mindfulness Questionnaire (FFMQ) [43], each capturing different aspects of interoception, hindering the development of consistent and objective assessment measures [44, 45]. Moreover, the relationship between components of IA, such as interoceptive accuracy and interoceptive sensibility, is not well-established, adding to the challenge of accurately quantifying IA [46, 47, 48].

Furthermore, research indicates that IA is crucial for self-awareness, recognising emotional states, and shaping subjective worldviews [6]. IA enables the brain to predict and address homeostatic needs, prompting necessary action [49]. Researchers have identified IA as a fundamental process that underpins various cognitive functions and significantly influences wellbeing [50]. Khalsa *et al*. [40] emphasise that enhancing our understanding of IA is essential, as it plays a pivotal role in mental health. The authors also suggest that IA can be shaped through learning, supporting the idea that MBIs may enhance IA over time, which could contribute to improvements in depressive symptoms [40].

### 1.4 Mindfulness-Based Interventions, Depression and Interoceptive Awareness

As interest in IA grows within psychological research, its connection to MBIs becomes clearer [51]. IA involves the perception and interpretation of internal bodily sensations, often considered a fundamental mechanism in mindfulness [37]. It plays a significant role in meditation practices by helping individuals stay present and cultivate equanimity [1, 52]. However, disruptions in IA can distort body awareness and impair emotional processes, exacerbating dysregulation [30, 53].

Research strongly associates disrupted interoception with mental health issues such as anxiety, depression, and eating disorders [36, 40, 54]. For example, individuals with depression often exhibit IA deficits compared to healthy controls [6]. Studies examining interoception in depression yield mixed results—some indicate that accuracy in heartbeat detection decreases as depressive symptoms worsen [55], while others suggest the opposite [56] or find no significant correlation [57]. Neuroimaging studies support the link between impaired IA and depression, showing reduced insula activity during interoceptive tasks in depressed individuals, suggesting that impaired IA may hinder effective emotion regulation [58, 59].

MBIs are increasingly recognised for their effectiveness in reducing depressive symptoms, with IA emerging as a crucial mechanism of improvement [6]. These interventions address IA deficits by enhancing decentring, leading to better treatment outcomes [6]. Khalsa *et al*. [40] emphasised that interoception is essential for physical and mental health, linking its dysfunction to various disorders. By improving IA, MBIs not only reduce depressive symptoms but also enhance treatment effectiveness [6].

While several systematic reviews have examined the effectiveness of MBIs in treating depression, none have specifically focused on the role of IA. This review aims to investigate the effectiveness of MBIs in reducing depressive symptomology and enhancing IA and explores whether IA mediates this relationship. Existing literature suggests a potential link between MBIs, IA, and depression, where increased IA, facilitated by MBIs, may reduce depressive symptoms. Therefore, it is hypothesised that:

H1: MBIs will lead to improvements in both depressive symptoms and IA.

H2: IA may act as a mediator in the relationship between MBIs and depressive symptoms.

## 2. Methods

The protocol for this systematic review was published in the International Prospective Register of Systematic Reviews (PROSPERO) before completion (CRD42023457300). This review was conducted and reported per the Preferred Reporting Items for Systematic Reviews and Meta-Analyses (PRISMA) checklist [60] (see **Supplementary Material**).

### 2.1 Search Strategy

In August 2024, a comprehensive literature search was conducted across the PsycINFO, MEDLINE, Scopus electronic databases. Additional studies were identified from Google Scholar and the reference lists of pertinent papers.

The included search terms were “mindfulness-based stress reduction*” or “MBSR” or “mindfulness-based cognitive therap*” or “MBCT” or “mindfulness-integrated cognitive behavioural therap*” or “mindfulness-integrated cognitive behavioral therap*” or “MiCBT” or “mindfulness-based intervention*” AND “interoceptive awareness” or ”interocept*” or “interoceptive attention” or “interoceptive accuracy” or “interoceptive sensibility” AND “depress*” or “depressive disorder” or “MDD” or “major depressive disorder”.

No date restrictions were applied. Studies published up to August 19th, 2024, were considered for inclusion.

### 2.2 Eligibility Criteria

Studies were included if they met the following criteria, structured using the PICO framework: (P) Adults aged 18 years and older; (I) MBIs explicitly derived from Mindfulness-Based Stress Reduction (MBSR), Mindfulness-Based Cognitive Therapy (MBCT), or Mindfulness-integrated Cognitive Behaviour Therapy (MiCBT); (C) Inclusion of a passive or active control/comparison group; and (O) Assessment of both depressive symptomatology and IA as primary outcomes, with IA measured subjectively using validated self-report tools pre- and post-intervention. Only quantitative studies with a minimum sample size of 20, published in English in a peer-reviewed journal, were eligible.

Studies were excluded if they involved participants under 18 years of age; used interventions not directly derived from MBSR, MBCT or MiCBT; lacked a comparison/control group; failed to assess both depressive symptoms and IA as primary outcomes, using subjective measures at both time points. Additional exclusion criteria included qualitative designs, sample sizes of fewer than 20, non-English language, and non-peer-reviewed formats (e.g., dissertations, case studies, pilot studies, systematic reviews, or meta-analyses).

### 2.3 Study Selection

**Fig. 1.**
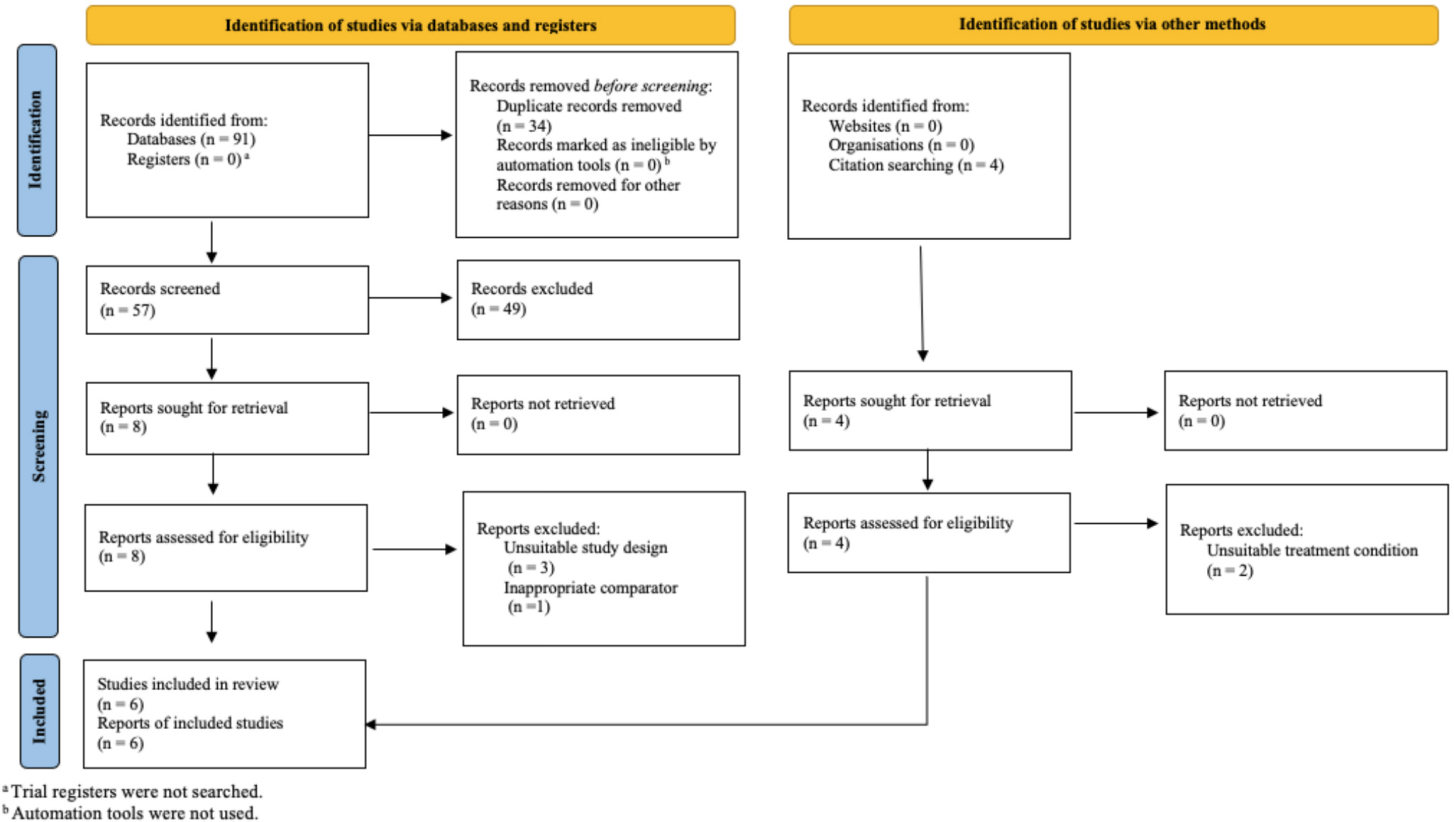
**Preferred Reporting Items for Systematic Reviews and Meta-Analyses (PRISMA) 2020 flow diagram showing the process of study identification, screening, eligibility assessment, and inclusion for the systematic review**.

The identified citations were imported into Covidence [61], a systematic review software, to streamline screening and extraction. Fig. 1 displays the PRISMA study selection flowchart based on eligibility criteria. After removing duplicates, the titles and abstracts of 61 studies were screened, excluding 49 unsuitable studies. Studies were deemed unsuitable if they did not include an MBI, did not measure depression or IA or were a pilot study, dissertation, systematic review or meta-analysis. Two independent reviewers conducted the screening process, achieving a 98.25% agreement rate. A full-text review of the remaining 12 studies led to the exclusion of six more due to unsuitable study design (*n* = 3), inappropriate comparator (*n* = 1), and unsuitable intervention (*n* = 2). The same two independent reviewers also performed the full-text review, with 100% agreement on the included studies. The final review included six studies that met the eligibility criteria.

### 2.4 Data Extraction

The following information was extracted and documented from the six included studies: (a) authors, year and country; (b) study design; (c) population of interest; (d) number of participants, mean age and standard deviation, (e) MBI applied and duration, (f) comparison/control condition, (g) outcome measures (depression and IA), (h) outcome measure findings, (i) mediation findings, (j) effect sizes (Cohen’s *d*) and (k) quality appraisal. This information is presented in Tables 1,2 (Ref. [6, 62, 63, 64, 65, 66]) , alphabetically by the first author’s last name.

**Table 1.** **Study characteristics and data extraction**.

Authors (year), country	Study design, population of interest	Participants (n), mean age^a^ (SD)	MBI chosen, intervention length	Comparator/control group	Outcome measures and findings	Mediation findings	Effect sizes (Cohen’s *d*)
Cascales-Pérez *et al*. [63]	Cluster-RCT	58	MBSR	Psychoeducation	Depression: POMS	NC	Depression: 0.45
Spain	Health professionals	Intervention: 52.36 (9.44)	8 Weeks		MBSR led to greater reductions in depressive symptoms over time compared to psychoeducation,		IA:
		Control: 49.64 (9.70)					*Observe*: 0.87
					*F* (1, 56) = 7.78, *p* < 0.01.		*Describe*: 0.28
					IA: FFMQ		
					MBSR showed greater improvement than psychoeducation on the FFMQ *Observe* subscale,		
					*F* (1, 56) = 12.15, *p* < 0.001. MBSR also led to greater increases in participants ability to observe their bodily sensations over time compared to psychoeducation,		
					*F* (1, 56) = 30.08, *p* < 0.001.		
Cascales-Pérez *et al*. [63] Spain					No statistically significant difference was found between MBSR and psychoeducation on the FFMQ *Describe *subscale, *F* (1, 56) = 1.65. However, MBSR led to greater changes in participants ability to describe their bodily sensations over time compared to psychoeducation, *F* (1, 56) = 6.13, *p* < 0.01.		
Fissler *et al*. [6]	RCT	74	MBCT	Restful activities	Depression: BDI-II	IA:	Depression: 1.27
Germany	Depressed population	Intervention: 42 (12.5)	3 Weeks		MBCT led to significantly lower depressive symptoms compared to restful activities (M_I–J_ = –9.6, *SE* = 1.8, *p* < 0.001).	*Body Listening *significantly mediated the relationship between MBCT and depressive symptoms (β = –0.76, *SE* = 0.22, *p* = 0.001).	IA:
		Control: 36.4 (12.5)					*Noticing*: 0.05
							*Not Distracting*: 0.40
							*Not Worrying*: 0.56
							*Attention Regulation*: 0.57
							*Emotional Awareness*: 0.16
							*Self-Regulation*: 0.86
Fissler *et al*. [6]					IA: MAIA	*Attention Regulation *(β = –0.25, *SE* = 0.13, *p* = 0.057), *Self-Regulation* (β = –0.09, *SE* = 0.14, *p* = 0.525) and *Trusting *(β = 0.08, *SE* = 0.13, *p* = 0.532) did not significantly mediate the relationship between MBIs and depressive symptoms.	*Body Listening*: 0.67
Germany					MBCT resulted in significantly higher IA across four subscales of the MAIA: *Attention Regulation* (M_I–J_ = 4.3, *SE* = 1.4, *p* = 0.004), *Self-Regulation* (M_I–J_ = 3.1, *SE* = 0.9, *p* = 0.001), *Body Listening* (M_I–J_ = –9.6, *SE* = 1.8, *p* = 0.008) and *Trusting *(M_I–J_ = 1.9, *SE* = 0.7, *p* = 0.01).		*Trusting*: 0.66
Karanassios *et al*. [64]	Cohort Study	112	MBSR + CBT	CBT	Depression: BDI	NC	Depression: 0.74
Germany	Depressed population	Intervention: 41.35 (11.2) Control: 40.85 (11.19)	4 Weeks		There was a significant reduction in depressive symptoms over time, *F* (1, 58) = 59.63, *p* < 0.001, indicating that both groups experienced a decrease in symptoms.		IA: 0.18
Karanassios *et al*. [64] Germany					However, no additional effect was found for the MBSR + CBT group compared to CBT alone, *F* (1, 58) = 0.34, *p* = 0.280.		
					IA: BPQ		
					Statistically significant improvements in IA were observed overtime, *F* (1, 58) = 3.79, *p* < 0.001. However, these improvements were not statistically significantly different between MBSR + CBT and CBT alone, *F* (1, 58) = 0.17, *p* = 0.341.		
Labelle *et al*. [65]	CCT	135	MBCR	WLC	Depression: POMS	NC	Depression: 0.48
America	Cancer patients	Intervention: 52 (11.00)	8 Weeks		The MBCR group exhibited a statistically significant reduction in depressive symptoms overtime.		IA:
		Control: 54 (10.90)					*Observe*: 0.75
							*Describe*: 0.45
Labelle *et al*. [65]					compared to the WLC group, *t* (209) = –3.95, *p* < 0.001.		
America					IA:		
					Statistically significant improvements in IA were found in the MBCR group compared to WLC on the *Observe*, *t *(209) = 6.34, *p* < 0.001, and *Describe*, *t* (209) = 4.58, *p* < 0.001, subscales of the FFMQ.		
Price *et al*. [66]	RCT	187	MABT	WHE or TAU	Depression: BDI-II	NC	Depression:
America	Women with substance use disorders	NR	8 to 10 Weeks		MABT results in statistically significant reductions in depressive symptoms compared to WHE and TAU (χ^2^ (2) = 13.51, *p* = 0.002).		MABT: 0.32 WHE: 0.25 TAU: 0.17
Price *et al*. [66]					IA: MAIA		IA:
America					MABT demonstrated statistically significant improvements in IA compared to WHE and TAU across the following MAIA subscales: *Noticing* (χ^2^ (2) = 13.51, *p* = 0.002), *Not Worrying* (χ^2^ (2) = 6.55, *p* < 0.04), *Attention Regulation *(χ^2^ (2) = 16.67, *p* < 0.001), *Emotional Awareness *(χ^2^ (2) = 12.46, *p* = 0.002), *Self-Regulation *(χ^2^ (2) = 14.75, *p* < 0.001), *Body Listening *(χ^2^ (2) = 9.99, *p* < 0.001), and *Trusting* (χ^2^ (2) = 18.18, *p* < 0.001).		*Noticing*:
							MABT: 0.64
							WHE: 0.64
							TAU: 0.75
							*Not Distracting*:
							MABT: 0.09
							WHE: 0
							TAU: 0.10
							*Not Worrying*:
							MABT: 0.30
							WHE: 0.20
							TAU: 0
							*Attention Regulation*:
							MABT: 0.67
							WHE: 0.61
							TAU: 0.48
							*Emotional Awareness*:
							MABT: 0.66
							WHE: 0.50
							TAU: 0.66
							*Self-Regulation*:
							MABT: 0.88
							WHE: 0.46
							TAU: 0.59
Price *et al*. [66]							MABT: 0.78
America							WHE: 0.66
							TAU: 0.57
							*Trusting*:
							MABT: 0.62
							WHE: 0.28
							TAU: 0.41
van der Velden *et al*. [62]	RCT	80	MBCT	TAU	Depression: QIDS-SR	NC	Depression: 0.81
America	Depressed population	Intervention: 43.17 (14.22) Control: 45.25 (12.01)	8 Weeks		MBCT statistically significantly reduced depressive symptoms compared to TAU (*p* = 0.001).		IA: *Noticing*: 0.94 *Attention Regulation*: 1.09 *Emotional Awareness*: 1.18 *Body Listening*: 0.99
					IA: MAIA		
					MBCT demonstrated statistically significant improvements in IA compared to TAU across the following MAIA subscales: *Noticing* (*p* < 0.001),		
van der Velden *et al*. [62] America					*Attention Regulation* (*p* < 0.001), *Emotional Awareness* (*p* < 0.001), and *Body Listening* (*p* < 0.001).		

*Note*. RCT, randomised control trial; CCT, controlled clinical trial; MBI, Mindfulness-based Intervention; MBSR, Mindfulness-based Stress Reduction; MBCT, Mindfulness-based Cognitive Therapy; MABT, Mindfulness Awareness in Body Oriented Therapy; MBCR, Mindfulness-based Cancer Recovery; CBT, Cognitive Behavioural Therapy; WLC, waitlist control; WHE, women’s health education; TAU, treatment as usual; POMS, Profile of Mood States; BDI-II, Beck Depression Inventory-II; QIDS-SR, Quick Inventory of Depressive Symptomatology–Self-Report; IA, Interoceptive awareness; FFMQ, Five Facets of Mindfulness Questionnaire; MAIA, Multidimensional Assessment of Interoceptive Awareness; BPQ, Body Perception Questionnaire; NR, not reported; NC, not calculated; SD, standard deviation; SE, standard error. ^a^Age reported in years.

**Table 2.** **Effective public health practice project quality assessment tool**.

Authors (year)	Selection bias	Study design	Confounders	Blinding	Data collection methods	Withdrawals and drop-outs	Global rating
Cascales-Pérez *et al*. [63]	1	1	2	2	1	1	Strong
Fissler *et al*. [6]	3	1	3	2	1	1	Weak
Karanassios *et al*. [64]	2	2	2	3	1	1	Moderate
Labelle *et al*. [65]	3	2	1	2	1	2	Moderate
Price *et al*. [66]	3	1	2	2	1	1	Moderate
van der Velden *et al*. [62]	2	1	2	2	1	2	Strong

Regarding (g), two studies used the FFMQ [43], which does not directly measure IA. Therefore, changes in the Observe and Describe subscales were extracted, as they most closely align with IA [67, 68].

### 2.5 Quality Assessment 

The Effective Public Health Practice Project tool (EPHPP) [69] was used to assess methodological quality and risk of bias across six areas: selection bias, study design, confounds, blinding, data collection, and withdrawals/dropouts. Sections were rated as strong (1 point), moderate (2 points), or weak (3 points). A global rating was assigned to each study as strong (no ‘weak’ rating), moderate (one ‘weak’ rating) or weak (two or more ‘weak’ ratings). This tool was chosen for its widespread use and sound reliability and validity [70].

## 3. Results

Six studies were included in this review (see Table 1). Sample sizes ranged from 58 to 187, with a total of 646 participants. Studies were undertaken in Germany (*n* = 2), Spain (*n* = 1) and America (*n* = 3). The study designs included three randomised control trials (RCT), one cluster RCT, one cohort study and one clinical control trial. Regarding population characteristics, studies included participants with depressive symptoms (*n* = 3), women with substance use disorders (*n *= 1), health professionals (*n* = 1) and cancer patients (*n* = 1). All studies included participants over 18 years, with the mean age across studies ranging from 36.4 to 54 years.

Diagnostic criteria for depression and baseline symptom severity scores were not consistently reported across the included studies. Where reported, symptom severity was generally described using self-report measures, but heterogeneous methods and reporting limited direct comparison across studies.

### 3.1 Mindfulness-Based Interventions

#### 3.1.1 Type of Mindfulness-Based Intervention 

Of the included studies, two utilised MBCT [6, 62], one employed MBSR [63], one incorporated a combination of MBSR and CBT [64], another applied Mindfulness-based Cancer Recovery (MBCR) [65] derived from MBSR, and the last used Mindful Awareness in Body-Oriented Therapy (MABT) [66], also adapted from MBSR.

#### 3.1.2 Length of Mindfulness-Based Intervention

The duration of MBIs ranged from 3 to 10 weeks. One study involved 90-minute sessions delivered weekly over 8 to 10 weeks [66], three studies were delivered weekly over 8 weeks [62, 63, 65], one study was bi-weekly for 4 weeks [64], and another had intensive 1.5-hour sessions weekly for 3 weeks [6]. Fissler *et al*.’s [6] intensive included daily home practice for 25 minutes twice daily, with participants receiving a psychoeducation booklet and a structured manual. All studies incorporated in-person mindfulness sessions accompanied by take-home meditation exercises.

### 3.2 Outcome Measures

#### 3.2.1 Depression

Regarding depression measures, three studies used the Beck Depression Inventory (BDI/BDI-II) [71], two utilised the Profile of Mood States (POMS) [72], and one employed the Quick Inventory of Depressive Symptomatology–Self-Report (QIDS-SR) [73].

#### 3.2.2 Interoceptive Awareness

Regarding IA measures, three studies used the MAIA [41], one utilised the BPQ [42], and two employed the FFMQ [43].

### 3.3 Effectiveness of Mindfulness-Based Interventions on Outcome Measures

#### 3.3.1 Significant Effect of Mindfulness-Based Interventions on Depression

All six included studies found that MBIs significantly improved depressive symptomology. According to Cohen’s [74] effect size interpretation, three studies reported a small effect of MBIs on reducing depressive symptoms [63, 65, 66]. Karanassios *et al*. [64] found a medium effect size, indicating that combining MBSR and CBT was moderately more effective than CBT alone. Notably, Fissler *et al*. [6] reported a large effect size, showing that MBCT was significantly more effective than restful activities in reducing depressive symptoms.

#### 3.3.2 Significant Effect of Mindfulness-Based Interventions on Interoceptive Awareness

Based on Cohen’s [74] effect size interpretation, all six studies found that MBIs significantly improved IA, though notable differences emerged across outcome measures. For example, Karanassios *et al*. [64] used the BPQ and reported minimal differences in IA abilities between MBSR and CBT compared to CBT alone.

Moreover, Cascales-Pérez *et al*. [63] found a large effect size for the *Observe* subscale of the FFMQ using MBSR, indicating substantial improvement in participants’ ability to observe internal bodily sensations relative to a psychoeducation group. Similarly, Labelle *et al*. [65] reported a large effect size for the same subscale using MBCR, suggesting an enhanced ability to observe bodily sensations compared to a waitlist control. Both studies noted small-to-moderate effect sizes on the *Describe* subscale, reflecting a slight-to-moderate increase in participants’ ability to describe internal sensations.

Regarding the MAIA, Fissler *et al*. [6] found small effects on the *Noticing*, *Not Distracting*, and *Emotional Awareness* subscales, moderate effects on the *Not Worrying*, *Attention Regulation*, *Body Listening* and *Trusting* subscales, and a large effect on the *Self-Regulation* subscale, indicating a strong impact of MBCT on self-regulation. In addition, Price *et al*. [66] reported small effect sizes for MABT on the *Not Distracting* and *Not Worrying* subscales and moderate effects on the Noticing, Attention Regulation, Emotional Awareness, Body Listening and Trusting subscales, with a large effect size for Self-Regulation, demonstrating significant improvement in self-regulation compared to controls. Additionally, van der Velden *et al*. [62] reported large effect sizes for the Noticing, Attention Regulation, Emotional Awareness, and Body Listening subscales in the MBCT group.

### 3.4 Mediating Role of Interoceptive Awareness

Fissler *et al*. [6] were the only study to explore IA as a mediator, finding that, among the facets of IA measured by the MAIA, only the *Body Listening* subscale mediated the relationship between MBCT and depressive symptoms.

### 3.5 Quality Assessment 

Table 2 presents the EPHPP quality assessment tool ratings. Two studies received a global rating of ‘strong’, three received a ‘moderate’ rating, and one received a ‘weak’ rating.

## 4. Discussion

The current systematic review aimed to examine the effects of MBIs derived from MBSR, MBCT, or MiCBT on reducing depressive symptoms and enhancing IA and to determine whether IA mediates this relationship. All included studies supported the first hypothesis, showing a significant effect of MBIs on both depressive symptomology and IA. The second hypothesis, proposing that IA may mediate the relationship between MBIs and depressive symptoms, was partially supported.

### 4.1 Synthesis of Results

Consistent with previous research, this review confirmed Hypothesis 1 that MBIs led to improvements in depressive symptoms [3, 8, 29] and IA [58, 59]. The reduction in depressive symptoms was expected, given the growing literature evaluating the efficacy of MBIs for alleviating depression [2, 30]. The relationship between MBIs and IA was also anticipated, as MBIs incorporate mindfulness and meditation techniques that enhance bodily awareness and promote emotion regulation and equanimity [1, 52].

Hypothesis 2 was partially supported, with Fissler *et al*. [6] being the only included study to identify IA as a mediating variable in the relationship between MBIs and depressive symptoms. Although MBCT significantly improved several aspects of IA as measured by the MAIA, the mediation effect was observed only for the *Body Listening* subscale. This indicates that enhancing the ability to listen to and interpret bodily sensations may be crucial in how MBIs contribute to reductions in depressive symptoms. However, due to study quality limitations, we currently cannot conclusively determine IA as a mediator across all aspects of interoception in this relationship.

Nonetheless, Fissler *et al*.’s [6] findings align with research suggesting IA as a mediator. For example, de Jong *et al*. [8] found that improvements in IA mediated the anti-depressive effects of MBCT in patients with comorbid depression and chronic pain. However, as a pilot study, it was excluded from this review based on our exclusion criteria. Similarly, Eggart and Valdés-Stauber [75] suggested that the *Self-Regulation* subscale on the MAIA partially mediated the relationship between depression and somatic symptom burden. Although not focused on MBIs, Eggart and Valdés-Stauber’s [75] findings are relevant, as this review found that self-regulation, a key IA component, had some of the largest effect sizes among the MAIA subscales (*d *= 0.86–0.88), indicating its importance in emotional wellbeing. Understanding the role of body listening in this context may inform future MBIs aimed at reducing depressive symptoms.

Notably, five studies did not investigate IA as a mediator [62, 63, 64, 65, 66]. This could be due to a focus on the main effects or direct relationships between variables, a lack of a theoretical framework for hypothesising mediators, or smaller sample sizes limiting the statistical power needed for mediation analyses [76]. Additionally, many studies lack detail on how much their MBI emphasises IA development, making it challenging to assess its role as a potential mechanism of change. This lack of precision may contribute to the under-exploration of IA as a mediator in current research. Future studies should clarify the techniques used to enhance IA within MBIs to better understand its role in mental health outcomes.

Additionally, substantial variation existed among the studies. While all employed MBIs, only Fissler *et al*. [6] and van der Velden *et al*. [62] used the same intervention, MBCT, but over 3 and 8 weeks, respectively. Although both studies used the MAIA, Fissler *et al*. [6] reported a large effect size on the *Self-Regulation* subscale, while van der Velden *et al*. [62] demonstrated large effect sizes for the *Noticing*, *Attention Regulation*, *Emotional Awareness*, and *Body Listening *subscales. The variability in effect sizes, duration, demographics, and control groups complicates comparisons, limiting the generalisability of findings and preventing strong conclusions about MBCT’s effectiveness across durations [77].

Beyond the observed variability in intervention protocols and outcome measurement approaches, the clinical populations examined across studies demonstrated considerable heterogeneity, including individuals with depression [6, 62, 64], cancer patients [65], women with substance use disorders [66], and healthcare professionals [63]. This heterogeneity limits the generalisability of findings and raises important considerations about the applicability of MBIs across diverse clinical contexts. Although preliminary evidence indicates benefits across populations, the mechanisms and magnitude of effect may differ between groups, highlighting the need for research into population-specific responses and tailored interventions.

MBSR and MBCT have consistently been shown to reduce depressive symptoms across various conditions [2, 10, 12]. However, differences in how these interventions cultivate IA may affect their specific therapeutic outcomes. This review found that MBIs derived from MBSR or MBCT effectively reduced depressive symptoms and enhanced IA. For example, MABT, derived from MBSR, was designed to teach core IA skills, such as recognising and evaluating bodily cues [37]. Using MABT, Price *et al*. [66] reported a large effect size for the MAIA’s *Self-Regulation* subscale, highlighting MABT’s effectiveness in enhancing self-regulation, which is closely linked to IA and emotional regulation processes [78]. Although Price *et al*. [66] found that MABT significantly improved IA, it only showed modest changes in depressive symptoms, suggesting that while it enhances IA, it may be less effective in targeting depressive symptoms compared to other interventions. These findings underscore the need to explore IA-focused approaches like MABT for their unique therapeutic potential, particularly in enhancing self-regulation and emotional wellbeing.

Furthermore, the outcome measures used to assess IA were heterogeneous. Half of the included studies employed the MAIA, the most widely used IA measure, which assesses various state-trait aspects of interoception [44]. However, flaws in the MAIA measure have been noted, such as weak associations between its *Not Distracting* and *Not Worrying* subscales and other MAIA subscales [79]. Moreover, two studies used the FFMQ, which has been criticised for only partially capturing IA in the *Observe* and *Describe* subscales [67, 68]. Additionally, neither the MAIA nor the FFMQ generates a total score, necessitating separate effect size calculations for each subscale. These factors, along with the limitations of this review, should be considered when interpreting its findings.

### 4.2 Strengths and Limitations

One strength of this review is its adherence to PRISMA guidelines for systematic reviews and preregistration on PROSPERO, which enhanced research quality and transparency [80]. The comprehensive search strategy ensured that a wide range of papers were screened for inclusion. Additionally, using two independent reviewers for screening reduced selection bias and enhanced accuracy and reliability [81]. Lastly, including studies with control or comparator conditions helped mitigate threats to internal validity [82].

Despite its strengths, this review has limitations that warrant caution in interpretation. A key limitation is the small number of included studies, attributed to the stringent search strategy, strict inclusion and exclusion criteria, limited available literature, and study heterogeneity, which prevented a meta-analysis. Furthermore, the quality of the included studies varied, with only two receiving a ‘strong’ rating, underscoring the limited quality of the available evidence.

### 4.3 Future Directions and Implications 

The current review’s findings suggest that MBIs have clinical implications for treating depressive symptoms as they are associated with increased IA and reduced symptoms. Future research should continue exploring IA as a key mechanism for optimising MBIs’ effectiveness in treating depressive symptoms.

Additionally, IA is often considered a mechanism of change in MBIs for reducing depressive symptoms; however, only one study in this review [6] specifically examined IA as a mediator. Kerr *et al*. [83] suggest that it remains unclear whether the focus on body sensations in MBIs directly contributes to these changes, highlighting the need for further research into the roles of IA and bodily awareness. MiCBT, which emphasises the development of equanimity through increased awareness of body sensations (i.e., IA), may offer valuable insights into how bodily awareness affects emotional regulation [5]. By teaching individuals to observe and become less reactive to bodily sensations, MiCBT could present a unique approach to alleviating distress and enhancing mental health. Rooted in ancient vipassana traditions, MiCBT employs body scanning techniques to cultivate awareness and equanimity, aiming to enhance insight into bodily sensations, manage distress, and reduce reactions that might perpetuate emotional suffering [5]. Although no included studies utilised MiCBT, its theoretical framework [36] and initial evidence support its effectiveness across various populations, including those with multiple sclerosis [34], diabetes [35] and comorbid anxiety and depression [84]. However, currently, there is no evidence that IA mediates the outcomes associated with MiCBT, as supporting data is lacking. Future research should explore IA’s role as a mechanism of change and examine the relationship between MiCBT, depression and IA, given its strong theoretical basis [36].

Although only one study in this review explicitly investigated IA as a mediator of treatment effects [6], employing a serial multiple mediation model with bootstrapping methods, it did not compare IA with other theoretically plausible mediators such as trait mindfulness or emotion regulation capacity. This focus on IA as a primary mediating factor limits conclusions regarding its unique contribution to therapeutic outcomes. Future research should consider using comparative mediation analyses that simultaneously test multiple potential mediators to clarify the relative influence of IA compared to other mechanisms underlying the therapeutic effects of MBIs.

A key challenge in advancing research into IA lies in its complex conceptualisation and measurement. IA encompasses objective measures, such as heart detection tests, and subjective scales, like the MAIA, BPQ, and FFMQ, which often include multiple facets beyond IA, blurring measurement boundaries. Objective measures provide insight into interoceptive accuracy (i.e., how well individuals detect internal signals), while subjective scales assess interoceptive sensibility, incorporating emotional and cognitive dimensions. This duality complicates comparisons across interventions, as each measure captures different aspects of interoception. Prior research has noted inconsistencies and concerns regarding the discriminant validity of the subjective IA measures included in this review [31, 45]. Thus, developing a widely adopted, psychometrically robust measure of IA is essential for improving diagnosis, treatment tracking, and understanding the mechanisms of change across conditions.

Despite these challenges, applying objective IA via the Mindfulness-based Interoceptive Exposure Task (MIET) [5, 85] offers a potentially valuable tool. Although not utilised in the included studies, the MIET is designed to expose participants to distressing sensations by observing them objectively rather than avoiding them [86]. This tool has shown promise in reducing pain, depression, and stress in chronic pain samples [87]. Further research is needed to explore its effectiveness in populations with depression and other groups where IA is crucial for therapeutic outcomes, such as those with eating disorders [88], post-traumatic stress disorder [89], and autism spectrum conditions [90]. As IA gains recognition as a transdiagnostic factor [91, 92], tools like the MIET may be instrumental in improving mental health outcomes. As Barrett and Quigley [50] suggest, interoception may hold the key to understanding health and illness, making it vital to investigate how tools like the MIET can help unlock the therapeutic potential of IA in diverse populations.

Lastly, this review’s lack of meta-analysis underscores the need for standardisation in future research, including uniform treatment manuals, consistent intervention durations, and standardised outcome measures. This would improve the reliability of findings, enable meaningful cross-study comparisons, and guide clinicians in making informed treatment decisions.

## 5. Conclusions

This review offers preliminary evidence supporting the potential of MBIs to reduce depressive symptoms and enhance IA. While the findings are suggestive of a mediating role for IA in these effects, current evidence is limited by small sample sizes, methodological variability, and high heterogeneity among study populations. These limitations constrain the generalisability of results and underscore the need for larger, well-controlled trials to verify these associations. As IA continues to gain attention as a possible transdiagnostic factor across mental health conditions, further research is warranted to clarify its therapeutic role within MBIs. Future studies should prioritise the systematic integration and measurement of IA to better understand its contribution to treatment outcomes and its potential utility across diverse clinical contexts.

## Availability of Data and Materials

The datasets generated and analyzed during the current study are available from the corresponding author on reasonable request.

## Supplementary Material

Supplementary material associated with this article can be found, in the online version, at https://doi.org/10.31083/AP39860.
